# The Impact of COVID-19 Related Changes on Air Quality in Birmingham, Alabama, United States

**DOI:** 10.3390/ijerph19063168

**Published:** 2022-03-08

**Authors:** Diya Jacob, Samuel Stowe, Iyinoluwa Babarinde, Aakruti Sharma, Abigail Christopher, M. J. Ruzmyn Vilcassim

**Affiliations:** 1 School of Health Professions, The University of Alabama at Birmingham, Birmingham, AL 35233, USA; diyaann@uab.edu (D.J.); ajc2020@uab.edu (A.C.); 2 Department of Environmental Health Sciences, School of Public Health, The University of Alabama at Birmingham, Birmingham, AL 35233, USA; gtostowe@uab.edu (S.S.); iyinbab1@uab.edu (I.B.); aakruti9@uab.edu (A.S.)

**Keywords:** air quality, COVID-19, particulate matter, nitrogen dioxide, Birmingham Alabama

## Abstract

Air pollution is responsible for a wide range of health effects in exposed populations. Variations in local air pollution can affect local population health outcomes. The strict regulations imposed during the peak of the COVID-19 pandemic (‘lockdowns’) resulted in a unique situation where human mobility was limited significantly, resulting in improved air quality in several major cities. The main goal of this study was to investigate if lockdowns during the COVID-19 pandemic significantly impacted air quality in Birmingham, Alabama—a city with a history of high air pollution levels—with a focus on PM_2.5_ (Particulate Matter with an aerodynamic diameter ≤2.5 µm) and NO_2_ (Nitrogen dioxide). Daily air pollutant and traffic data were obtained for the Birmingham Metropolitan Area for the period January to October 2020, and previous years. Mean PM_2.5_ and NO_2_ concentrations and traffic volumes during the official city/state lockdown period (24 March to 30 April 2020) were compared to pre- and post-lockdown means. The mean PM_2.5_ and NO_2_ concentrations during the lockdown did not significantly differ from that of the pre- or post-lockdown periods. However, NO_2_ significantly decreased even after the lockdown order was removed, with the mean decreasing significantly compared to pre-lockdown and lockdown periods. Both PM_2.5_ and NO_2_ annual means in 2020 were significantly lower than the annual means in 2019, indicating the occurrence of significant changes over the longer term that were not limited by defined lockdown periods. Traffic significantly increased after the lockdown order was removed but did not correlate with the two pollutants studied. Therefore, we conclude that the Stay at Home/lockdown regulations and other COVID-19 restrictions had an impact on the air quality of Birmingham Alabama; although these lockdown impacts varied for each pollutant and were not limited only by the official lockdown dates/periods.

## 1. Introduction

Air pollution is responsible for causing a range of adverse health effects including, respiratory and cardiovascular diseases, chronic airway diseases, lung cancer, and increased mortality [1,2,3,4,5]. The World Health Organization estimates that air pollution related diseases are responsible for over 7 million deaths every year, of which 4.2 million deaths are attributed to outdoor air pollution [5]. The six most common criteria pollutants that are associated with most of these adverse health effects include Particulate Matter, Nitrogen Dioxide (NO_2_), Carbon Monoxide (CO), Sulfur dioxide (SO_2_), Ground-level Ozone (O_3_), and Lead (Pb) [6]. Particles with an aerodynamic diameter ≤ 2.5 µm are particularly harmful as they can penetrate deeper into the lungs, and therefore, is important in the study of air pollution related health outcomes. The gaseous pollutants are also associated with a range of harmful health effects impacting the respiratory system, mainly as irritants. In the environment, they can act as precursors in the formation of secondary pollutants including Ozone and secondary particles [7].

Various chemical constituents released from diverse sources combine to form the PM_2.5_ pollutant components of a city/region. Therefore, the study of PM_2.5_ and related public health outcomes can be complex. Source apportionment studies have demonstrated that the major sources of PM_2.5_ in US cities were metal industries, crustal/solid particles, Motor Vehicles, Steel Industries, Coal Combustion, Salt Particles, and Biomass burning [7]. The primary source of NO_2_ emission was found to be vehicular traffic, although industrial processes can also be a significant contributor. SO_2_ was most correlated to Oil/Diesel and coal combustion. While stationary and mobile anthropogenic sources (industrial and traffic emissions) can contribute significantly to air pollution in cities [8], natural phenomena, such as dust storms and smog events can also significantly alter air quality [9]. Emissions from local stationary and mobile sources significantly impact local air quality; changes in traffic volumes, variations in emissions from industries and power plants, and weather patterns contribute to short- and long-term air quality variations. For example, during the Beijing Olympics in 2008, authorities in Beijing implemented restrictions on industry operations and changed traffic plans, resulting in improved air quality over the short term [10]. Studies have shown that exposure to such varying air quality impacts health outcomes, and improved air quality can result in reductions in adverse outcomes [11,12,13,14]. While exposure to short-term elevated levels of PM can result in lowered lung function, subsequent improvement in air quality has been shown to be associated with improvements in lung function and reduction in respiratory symptoms (i.e., possible ‘recovery’) [15].

In March 2020, a major change in human mobility was caused by the restrictions imposed to control the Severe Acute Respiratory Syndrome Coronavirus 2 (SARS-CoV-2), or the COVID-19 pandemic [16,17]. In order to contain the spread of the virus, many states in the U.S. and countries around the world-imposed restrictions and guidelines, such as lockdowns, social distancing rules, travel bans, and limited business operations. This in turn caused direct and indirect effects on air quality across several countries, including the U.S. [18]. Current findings demonstrate that, overall, the pandemic resulted in improved air quality in most major cities. Major cities with populations of more than 1 million, such as Los Angeles, Chicago, Phoenix, Philadelphia, San Antonio, San Jose, New York, and San Diego saw PM_2.5_ concentrations decrease significantly compared to the previous year’s values during the same period [19,20], while some cities had drops in concentrations compared to previous months [18,21,22,23]. Data collected from over 2000 monitoring sites located in USA, Canada and Mexico demonstrated that gaseous pollutants, such as NO_2_ had significantly decreased in April 2020 in comparison to the month of April in the past five years [24]. This decrease in NO_2_ was directly linked to locations that witnessed a reduction in mobility. In contrast, PM_2.5_ concentrations across most sites in this study witnessed no significant change but rather, saw an increase in some of the sites [24]. Studies in other parts of the world have also demonstrated mixed results. Despite the expectation of a significant reduction in air pollutants due to the lockdowns, not all urban areas experienced reduced levels. During the initial period of the lockdown in China (January–February 2020), some regions saw an increase in PM_2.5_. Levels of PM_2.5_ in Northern China rose compared to other regions, possibly due to severe haze events. During this time, a mean increase of 30.6 µg/m^3^ was reported compared to the previous year [25]. London and Paris both reported increased levels of PM_2.5_ during the quarantine period [26]. During the period from 24 March to 31 May 2020, in Delhi, India, it was found that PM_2.5_, NO_2_ and CO had reduced by 47%, 68% and 58%, respectively, and a significant increase in O_3_ when compared to 2019 [27]. North India as a whole experienced a 34% decrease in PM_2.5_ concentration compared to 2019, however, O_3_ concentrations increased [23]. Ozone in New Delhi increased temporarily (during April–May) compared to the previous lockdown phase. Some studies concluded that air quality had improved during the lockdown period with each pollutant varying in its concentration in relation to the lockdown irrespective of the geographical and climatic conditions [28]. However, when studying the impact of lockdowns on air quality, factors, such as seasonal changes and the impact of meteorological events on air pollution have to be considered [29]. 

Historically, Birmingham—the largest and most populous city in Alabama, was known to have one of the worst air quality levels in the country [30]. The major contributors were mines, industrial mills, and factories that manufactured iron ore. Early studies during the peak of the manufacturing boom in Alabama pointed out the importance of industrial emissions on Birmingham’s air quality, which identified domestic, transportation, commercial, and industrial sources as predominant sources [31]. A study conducted in 1956 during and after the steel strike in Birmingham, AL showed the effect of the industries on particulate matter [32]. The average level of suspended particulate matter increased significantly once the steel industries resumed production. Elevated levels of air pollution in Birmingham were shown to be associated with increased daily mortality and increased hospital admissions for the elderly [33,34]. However, in recent times air quality in Birmingham has improved dramatically, although still among the 15 most polluted cities in the US (based on PM levels from 2016 to 2018) as per a recent ‘State of the Air’ report by the American Lung Association [35]. The metropolitan Birmingham area ranked 14th in the nation for year-round PM pollution, however, was the best ever from the years 2016–2018, and met the United States Environmental Protection Agency (US EPA) standards during this period. Notwithstanding this drastic change and improvement in air quality, recent studies on criteria pollutant concentrations in Birmingham, AL are very limited. Importantly, at the time of the submission of this manuscript, we are not aware of any peer-reviewed studies that have researched the change in levels of criteria pollutants in Birmingham, AL. Given the history of air pollution and being the largest city in Alabama, we believe a detailed study of how COVID-19 related changes impacted air quality in the city is important and will contribute to other studies investigating health impacts. 

Therefore, the primary objective of this study was to investigate the changes in selected criteria air pollutant levels in the Birmingham Metropolitan Area during the COVID-19 pandemic period, in comparison to ‘Business as usual’, i.e., previous months and years prior to the pandemic. The study primarily focused on PM_2.5_ and NO_2_ levels as they were considered markers of mobile source air pollution, which was impacted by the restrictions.

## 2. Materials and Methods

Our study utilized regional-level air pollutant and traffic data to investigate if air pollutant levels were significantly impacted during the government ‘Stay at Home Order’ (i.e., COVID-19 lockdown) period in Birmingham, Alabama, USA. Primary air pollutants of focus were PM_2.5_ (Particulate matter with diameter less than 2.5 µm) and NO_2_ (Nitrogen dioxide), although data were collected and limited analyses were conducted for other criteria pollutants including Ozone (O_3_), Carbon monoxide (CO) and Sulfur dioxide (SO_2_). The official ‘lockdown’/Stay at Home Order period for the city of Birmingham (AL) went into effect on the 24 March 2020 at 12.00 PM [36], followed by a state-wide lockdown (Alabama) from 4 April to 30 April 2020 [37,38]. However, some restrictions remained in effect until mid-May, and businesses were permitted to open for regular operation only on 22 May 2020. For analyses in this study, we defined pre-lockdown, lockdown and post-lockdown periods as 17 February to 24 March, 25 March to 30 April and 1 May to 6 June, respectively. 

### 2.1. Data Sources and Measurements

#### 2.1.1. Air Quality Data Retrieval

**Figure 1:**
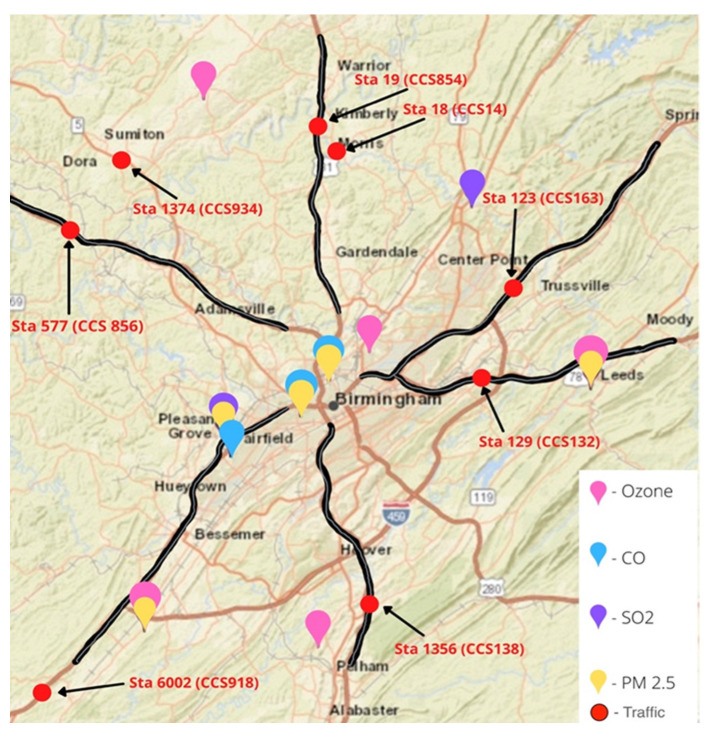
Map of the Birmingham Metropolitan area and the air and traffic monitoring sites used in this study. Highways where traffic monitoring sensors are located are highlighted. Map source: US EPA Interactive Map of Air Quality Monitors.

Daily concentration data for selected criteria pollutants were obtained for the period January 2016 to October 2020, from US EPA, fixed air quality monitors via the EPA Air Quality System (AQS) database for Birmingham, Alabama. The EPA Air Quality System is a platform where air quality data is collected from around the country, and publicly available to download. Our study primarily evaluated PM_2.5_ and NO_2_ variations in the Birmingham Metropolitan Area located in Jefferson County, Alabama. Table 1 contains details on air pollutants, number of monitoring sites, and measurement metrics analyzed in this study, and Figure 1 shows the locations of the monitoring stations. Please see supplemental Table S1 for a list of all monitoring sites that data were obtained from, for the study.

#### 2.1.2. Traffic Data Retrieval

The daily traffic data for Jefferson County, Birmingham was downloaded from the Alabama Department of Transportation (ALDOT) for 2019 and 2020. The data collected reflected the total amount of vehicles that entered and exited Jefferson County on a given day in 2019 and 2020. To estimate the traffic that entered and exited Jefferson County, data were obtained from traffic monitoring sites located (1) Along eight major highways/interstate routes where traffic enter/exit Birmingham and (2) from two sites closest to the respective central air monitors in Birmingham (Figure 1). The totals and averages of inbound and outbound traffic volumes were calculated and analyzed.

#### 2.1.3. Data Processing

Some central monitor sites had up to three different monitors located in close proximity with the same site ID. In such cases, the average of these sub-station values was taken as the site’s concentration. If there was no traffic data provided for a given day, the average of the day before and after was used. If the traffic data was not provided for more than a week to a month, the average of the previous two months was used.

### 2.2. Statistical Analysis

Daily mean values of pollutants were used to calculate descriptive statistics including the mean monthly concentrations and Standard Deviations, (SD) with more detailed data and analyses focusing on PM_2.5_ and NO_2_. A General Linear Model Analysis of Variance (ANOVA) was employed to analyze the mean concentration differences between pre, during and post lockdown periods and monthly concentration differences between months in 2020. Tukey’s simultaneous tests were used to compare the differences in means post ANOVA. Student’s t-tests were used to compare each month’s concentration in 2020 with the corresponding months in 2019. A linear regression analysis was conducted to analyze the correlation between traffic volumes and PM_2.5_ and NO_2_ concentrations. A significance level of *p* ≤ 0.05 was considered for all analyses. Analyses were completed using the Minitab Version 19 statistical software (Minitab LLC, State College, PA, USA), which was also used to generate figures. Some figures were generated using Microsoft Excel version 16.16.27 (Microsoft, Redmond, WA, USA).

## 3. Results

### 3.1. Descriptive Statistics

Descriptive statistics for each air pollutant, and traffic volumes, in 2019 and 2020 are shown in Table 2. For some of the pollutants, data were not available for the full year of 2019 or 2020 at the time they were collected, and therefore, the data used to generate the descriptive statistics for each pollutant spans to the latest date available at the time of data collection. In 2020, Birmingham experienced a dust storm from 26–28th June, which was considered an outlier for PM_2.5_ concentration statistics (the outlier values were over 4 times the mean and confirmed by an outlier test in Minitab). Mean PM_2.5_ concentrations with and without the outliers are reported.

Briefly, PM_2.5_ concentrations in 2020 during the study period (January to October) ranged from 3.1 to 36.9 µg/m^3^, while the mean PM_2.5_ concentration (including the outliers in June) for that year was 9.1 µg/m^3^ with a standard deviation of 3.6 µg/m^3^. When the outliers were removed, the mean PM_2.5_ concentration was 8.9 µg/m^3^ with a standard deviation of 3.4 µg/m^3^. For 2020, the NO_2_ data available ran from January to August from the two NO_2_ monitoring sites (North Birmingham and Arkadelphia), during which time the average NO_2_ concentration was 20.76 ppb with a standard deviation of 8.29 ppb. NO_2_ concentrations during this period ranged from 5.45 to 44.15 ppb. Traffic volumes during the study period varied widely from a minimum of 98,484 to 494,507 vehicles, with a mean and standard deviation (SD) of 347,888 and 70,922, respectively.

We also calculated means and SDs during the lockdown period (38 days), pre and post lockdown periods (38 days before the lockdown and 38 days after the lockdown was lifted, respectively) including the post lockdown period after businesses opened, which are shown in Table 3.

### 3.2. Variations in PM_2.5_ and NO_2_ Concentrations during the Study Period and Comparisons with Previous Years

**Figure 2:**
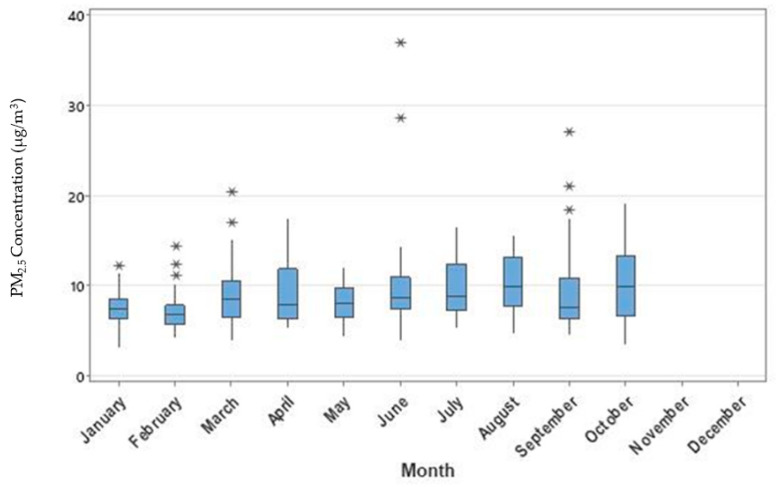
Boxplot of monthly PM_2.5_ concentrations (µg/m^3^) in 2020 (January–October). The * symbol represents values that were considered outliers for that particular month.

**Figure 3:**
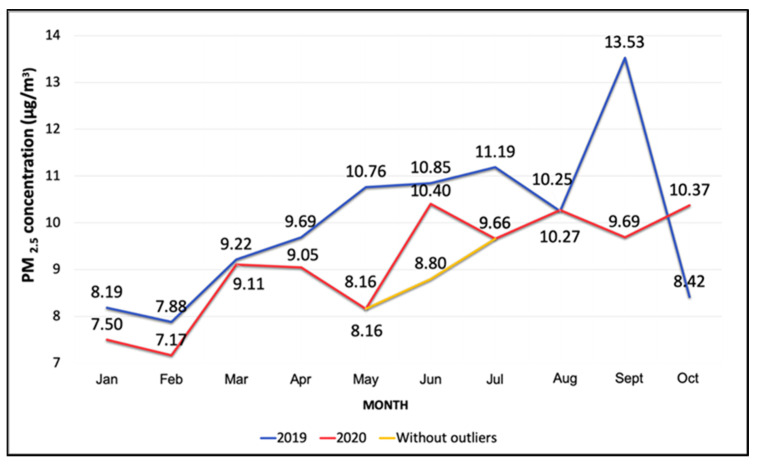
Variation in monthly mean PM_2.5_ concentrations (µg/m^3^) from January to October in 2019 and 2020. The mean concentration in June is shown with and without the outlier due to a dust storm.

**Figure 4:**
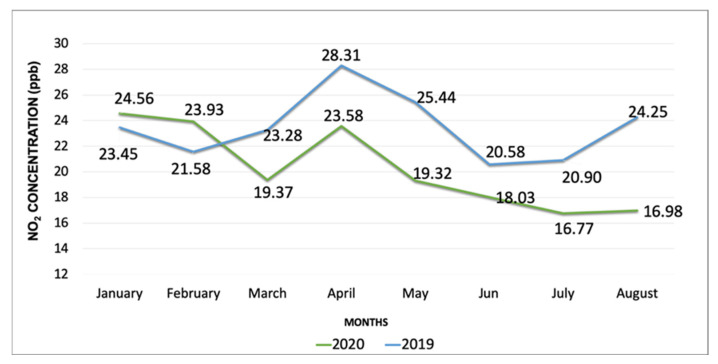
Variation in monthly mean NO_2_ concentrations (ppb) from January to August in 2019 and 2020.

**Figure 5:**
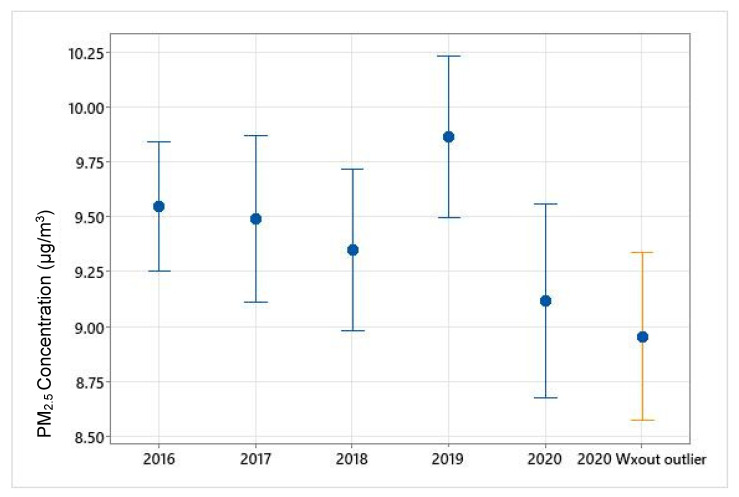
Yearly mean PM_2.5_ concentrations from 2016 to 2020. Error bars represent 95% confidence interval of the mean.

Monthly variations in PM_2.5_ concentrations during the study period (January–October 2020) are shown in Figure 2 and Figure 3. PM_2.5_ concentrations gradually decreased from March through May, however, increased throughout the rest of the months until October. The boxplot (Figure 2) demonstrates the variations within each month and outliers. NO_2_ concentrations saw a temporary increase in April compared to March, however, monthly mean values continued to decline from April to August 2020 (Figure 4). Overall, the mean PM_2.5_ and NO_2_ concentrations in 2020 were statistically significantly lower compared to the mean concentrations of these pollutants in 2019 (Figure 5 and Supplemental Figure S1, respectively). The mean PM_2.5_ concentration of 2020 was also the lowest compared to historical yearly averages from 2016 to 2019 (Figure 5). PM_2.5_ concentrations of 2019 and 2020 by month are shown in Supplemental Figure S2. 

### 3.3. Comparison of Air Quality and Traffic Volumes between Lockdown, Pre-Lockdown and Post-Lockdown Periods

**Figure 6:**
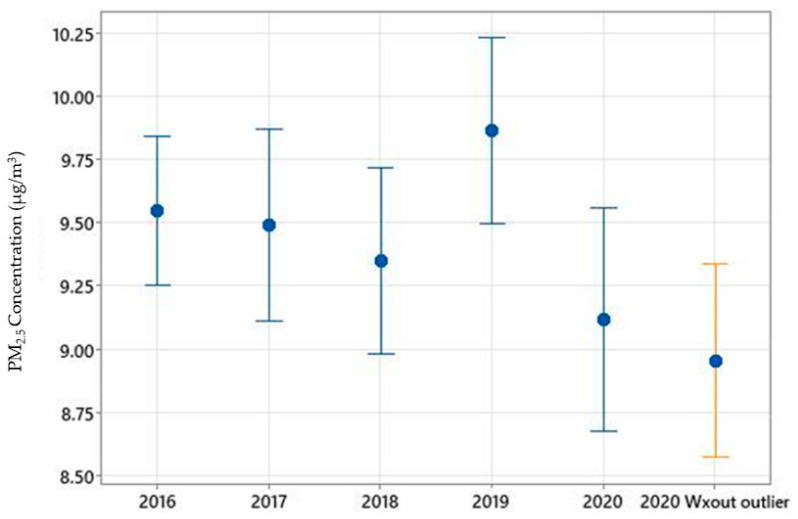
Change in mean PM_2.5_ concentrations before, during, and after the lockdown period. Error bars represent 95% confidence interval of the mean. Abbreviations: PRE LD: Pre-lockdown, LD: Lockdown, POST LD: Post-lockdown and POST LD_B OPEN: Post-lockdown—businesses open.

**Figure 7:**
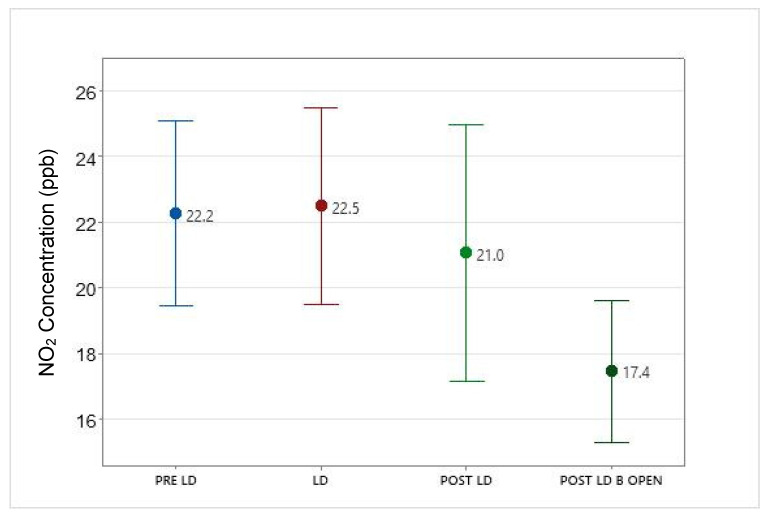
Change in mean NO_2_ concentrations before, during, and after the lockdown period. Error bars represent 95% confidence interval of the mean. Abbreviations: PRE LD: Pre-lockdown, LD: Lockdown, POST LD: Post-lockdown and POST LD_B OPEN: Post-lockdown—businesses open.

Mean PM_2.5_ concentrations recorded during the pre-lockdown, lockdown and post-lockdown periods were 8.3, 9.3, and 8.2 µg/m^3^, respectively (Table 3 and Figure 6). Post-ANOVA comparisons of PM_2.5_ concentrations between the lockdown and non-lockdown periods did not identify any significant differences between the lockdown period and pre and post lockdown periods. However, significant differences in NO_2_ concentrations between these periods were identified. While the mean NO_2_ concentration did not significantly decrease due to the lockdown (compared to pre-lockdown), surprisingly, the mean NO_2_ level reduced significantly during the post-lockdown period/s compared to the lockdown period (*p* ≤ 0.05) (Figure 7). Therefore, our data indicate that NO_2_ concentrations did not go down significantly during the official city/state lockdown period but continued to reduce after the stay at home orders were removed. Table 3 shows the mean values of each pollutant during these periods.

**Figure 8:**
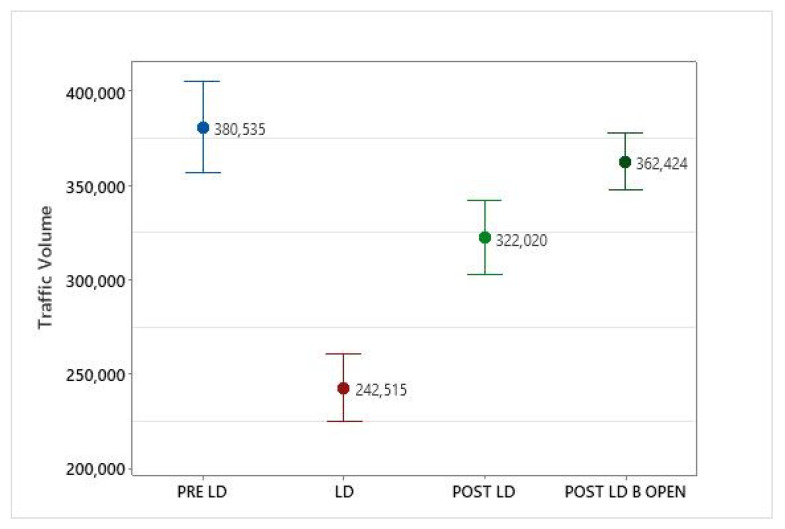
Change in traffic volumes before, during, and after the lockdown period. Error bars represent 95% confidence interval of the mean. Abbreviations: PRE LD: Pre-lockdown, LD: Lockdown, POST LD: Post-lockdown and POST LD_B OPEN: Post-lockdown—businesses.

As expected, traffic levels significantly reduced during the lockdown period compared to pre-lockdown (*p* ≤ 0.05) and increased significantly during the post-lockdown period (Table 3). However, the average traffic volume in the post-lockdown period did not reach pre-lockdown levels and was significantly lower (*p* ≤ 0.05) than the pre-lockdown level (Figure 8). Post-ANOVA Tukey comparisons identified that the mean traffic volume during the lockdown period was statistically significantly lower compared to mean levels of all other periods analyzed (i.e., Pre-lockdown, post-lockdown including after businesses were open).

Further analyses on the impact of traffic on air pollutant levels were conducted using linear regression analysis. However, we did not find significant correlations between PM_2.5_ concentrations or NO_2_ concentrations with traffic volumes, either when analyzed with the total volumes (Northbound + Southbound total) or with the average traffic volume (*p* = 0.062, R^2^ = 1.4 and *p* = 0.523, R^2^ = 0.2 for PM_2.5_ and NO_2_, respectively). 

## 4. Discussion

To our knowledge, this is the first study that investigated the impact of COVID-19 related lockdowns on the air quality of the Birmingham City/ Metropolitan Area, a city with a history of elevated air pollution levels. Our findings primarily demonstrate that mean PM_2.5_ and NO_2_ concentrations in Birmingham, AL in 2020 were significantly impacted compared to the previous year, however, levels during the official lockdown period in 2020 were not significantly reduced, compared to pre-lockdown levels. Although NO_2_ concentrations did not decrease during the official lockdown, NO_2_ concentrations significantly declined even after the lockdown order was removed (i.e., during the post-lockdown period), indicating that COVID-19 related changes did significantly impact air quality in Birmingham, AL over the following months. Results from past studies on the impact of lockdowns on air quality in cities are mixed, with some reporting similar results as this study with PM and/or NO_2_ concentrations [39], and others reporting significant increases or decreases in PM and other gaseous pollutant concentrations due to lockdowns in various cities [18,20,40,41]. Overall, the average PM_2.5_ and NO_2_ yearly mean concentrations in 2020 were significantly lower compared to the annual means of these pollutants in 2019, demonstrating that when lockdown and post-lockdown periods are combined, the COVID-19 related changes possibly impacted yearly mean air pollutant levels in Birmingham, AL. 

When inferring changes in air quality due to COVID-19 related lockdowns, it is important to also consider the ‘baseline’ air pollutant levels of a city. Most studies that have reported significant/drastic reductions in PM and other pollutants were in cities with high air pollution levels, such as cities in India, China and Brazil [23,40,42]. We assume that in cities that have relatively lower mean PM and NO_2_ annual means, such as Birmingham during this period (which are in attainment to EPA standard levels—see Table 1), such significant changes may not be observed [39]. Studies have demonstrated that the contributing factors to air quality changes during the COVID-19 lockdowns are complex and vary depending on urban/commercial vs. rural neighborhoods, primary energy sources used (wood vs. coal) and influence of secondary pollutants, such as Ozone in some regions [43,44,45]. 

As expected, traffic volumes significantly reduced during the lockdown, and increased back to significant levels (although not reaching pre-lockdown levels) during the post-lockdown periods. However, this change was not correlated with the changes in the two main air pollutants studied during the defined periods. As traffic is a major contributor NO_2_, it is possible that traffic volume reduction contributed to post-lockdown reductions in NO_2_. We also assume that ‘Limited Business Operations’ imposed early by the largest employer of the city—The University of Alabama at Birmingham—impacted traffic and stationary source emissions before the official lockdown periods, and therefore, affected the concentrations of the pre-lockdown and during lockdown periods. The presence of busy ‘satellite’ suburban areas surrounding Birmingham city may have also contributed; while traffic volumes that were entering Birmingham city reduced, mobility in surrounding suburban areas were likely not reduced.

The air pollutant and traffic data showed mild to moderate skewness, and therefore, we performed the analyses using non-parametric tests such as the Kruskal–Wallis test, to compare levels of pre-, during, and post-lockdown periods. Results from the non-parametric tests revealed the same outcomes.

The study had several limitations. We relied on regionally available EPA data which had limitations with regards to the number of monitoring sites as well as data availability. While sufficient central monitors were present to measure PM_2.5_ concentrations in the Birmingham Metropolitan Area, only two NO_2_ monitoring sites were present, which may not represent the levels in areas that are further away from these two monitors. This limitation in central sites also limited our ability to analyze variations in other criteria pollutants, such as Ozone. Additionally, we were unable to factor in the contributions from stationary sources on the concentration variations of these pollutants. Birmingham and surrounding areas are still home to many industries, and it is possible that emissions from powerplants and other stationary sources were impacted during the lockdown periods, with some possibly increasing activity and emissions. 

## 5. Conclusions

Our study found that air quality in Birmingham, AL significantly improved in the months studied in 2020, compared to the previous year (2019). However, PM_2.5_ concentrations did not show significant variations between the defined lockdown period and the pre- and post-lockdown periods, which were based on the city and state-wide ‘stay at home’ orders that significantly reduced mobility and traffic volumes. NO_2_ mean concentrations were significantly impacted, although not during the official lockdown period. The significant reduction in the post lockdown NO_2_ mean indicates that COVID-19 related changes had a significant impact on air quality. However, both pollutant concentration variations did not correlate with traffic volumes entering and exiting the Birmingham city area. Further studies on the impact and interaction of mobile and stationary sources, and concentration variations of secondary pollutants, such as Ozone, can improve our understanding of how COVID-19 related changes or other major events impact a city’s air quality.

## Figures and Tables

**Table 1:** Air pollutants, number of monitoring stations and related metrics used in the study.

Pollutant	Unit	Concentration Parameter	Number of Central Monitor Sites Data Retrieved from	Average Concentration in 2020 (January–October)	EPA NAAQS ^a^ (Primary)
PM_2.5_	µg/m³	Daily Mean	7–8	9.1	12 (annual)
NO_2_	ppb	Daily Maximum1-h	2	20.76	53 (annual)
O_3_	ppm	Daily Maximum 8-h	7	0.040	0.070
SO_2_	ppb	Daily Maximum1-h	2	1.70	75
CO	ppm	Daily Maximum 8-h	3	0.39	9

^a^ National Ambient Air Quality Standards.

**Table 2:** Descriptive statistics of air pollutant concentrations (of interest in this study) and traffic volumes in 2019 and 2020 in Birmingham, Alabama.

		Mean (±Standard Deviation)		Minimum		Maximum		(*n*) Days	
	Year	2019	2020	2019	2020	2019	2020	2019	2020
Metric									
PM_2.5_ (µg/m^3^)		9.9 (±3.6)	9.1 (±3.9) 8.9 (±3.4) ^b^	2.9	3.1	20.0	36.9	365	297
NO_2_ (ppb)		24.58 (±10.39)	20.76 (±8.29)	5.45	5.35	64.05	44.15	360	243
O_3_ (ppm)		0.044 (±0.01)	0.040 (±0.01)	0.017	0.015	0.075	0.063	244	245
SO_2_ (ppb)		4.90 (±4.11)	1.70 (±1.45)	0.33	0.15	26.55	10.9	365	366
CO (ppm)		0.43 (±0.19)	0.39 (±0.19)	0.1	0.1	1.25	1.1	365	366
Traffic: Number of vehicles		409,405.6 (±52,166.8)	347,887.6 (±70,921.5)	246,023	98,484	504,150	494,507	243	244

^b^ PM_2.5_ mean and SD without the outliers in June 2020.

**Table 3:** Mean and standard deviation of PM_2.5_ and NO_2_ concentrations, and traffic volumes before, during and after the lockdown period in Birmingham, AL.

Parameter	Pre-Lockdown (17 February–24 March)	Lockdown (25 March–30 April)	Post-Lockdown (1 May to 22 May)	Post-Lockdown (Businesses Open—22 May–30 June)
PM_2.5_ (µg/m^3^)	8.3 (±3.4)	9.3 (±3.4)	8.2 (±2.0)	8.6 (±2.4)
NO_2_ (ppb)	22.2 (±8.4)	22.5 (±8.8)	21.0 (±8.6)	17.4 (±6.8) *
Traffic	380,535 (±72,972)	242,515 * (±53,389)	322,020 (±42,809)	362,424 (±46,327)

* Indicate means that were statistically significantly different from all other means in their respective categories (*p* < 0.05).

## Data Availability

Air pollutant data used in this study can be retrieved from the United States Environmental Protection Agency (US EPA) website: www.epa.gov.

## Supplementary Materials

The following are available online at https://www.mdpi.com/article/10.3390/ijerph19063168/s1, Figure S1: Annual mean NO_2_ concentrations (ppb) from year 2016–2020. Error bars represent 95% confidence interval of the mean, Figure S2: Monthly mean PM_2.5_ concentrations (μg/m^3^) in years 2019 and 2020 (Error bars represent 95% confidence interval of the mean), Table S1: Locations of central air monitoring sites that provided data for the study.
