# Supplementary material for: The Impact of COVID-19 Related Changes on Air Quality in Birmingham, Alabama, United States
Source: Int J Environ Res Public Health. 2022 Mar 8;19(6):3168. doi: 10.3390/ijerph19063168 (PMC8951610; doi:10.3390/ijerph19063168)
Supplement: Supplementary file 1 [file ijerph-19-03168-s001.zip › ijerph-1615175-supplementary.pdf]

Supplementary Material

1. Supplemental Tables

US EPA central air monitoring site locations in Birmingham, Alabama (Jefferson County and nearby suburbs) where pollutant data were obtained are given below.

Table S1: Locations of central air monitoring sites that provided data for the study

Sl. No	Pollutant	Sites 2019	Sites 2020
1	$PM_{2.5}$	-North Birmingham<br>-Mc Adory<br>-Leeds<br>-Wylam<br>-Arkadelphia<br>-Corner<br>-Sloss Shuttlesworth	-North Birmingham<br>-Mc Adory<br>-Leeds<br>-Wylam<br>-Arkadelphia<br>-Corner<br>-Sloss Shuttlesworth
2	NO_2	-North Birmingham<br>-Arkdelphia	-North Birmingham<br>-Arkdelphia
3	O_3	-North Birmingham<br>-McAdory<br>-Fairfield<br>-Leeds<br>-Corner<br>Terrant Elementary School<br>-Helena	-North Birmingham<br>-McAdory<br>-Leeds<br>-Corner<br>-Terrant Elementary School<br>-Helena
4	CO	-North Birmingham<br>-Fairfield<br>-Arkadelphia	-North Birmingham<br>-Fairfield<br>-Arkadelphia
5	SO_2	-North Birmingham<br>-Fairfield	-North Birmingham<br>-Fairfield

2. Supplemental Figures

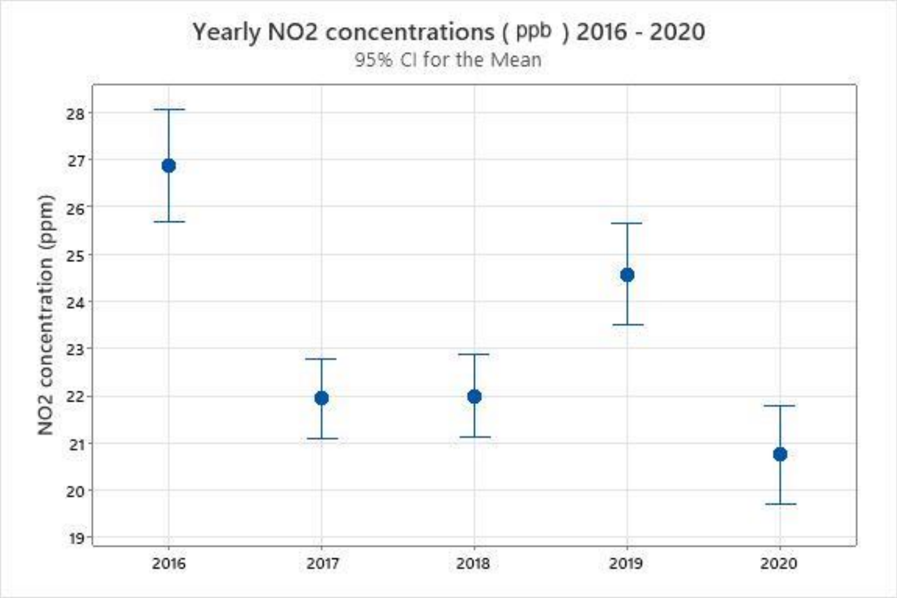

Figure S1: Annual mean NO_2 concentrations (ppb) from year 2016 – 2020. Error bars represent 95% confidence interval of the mean

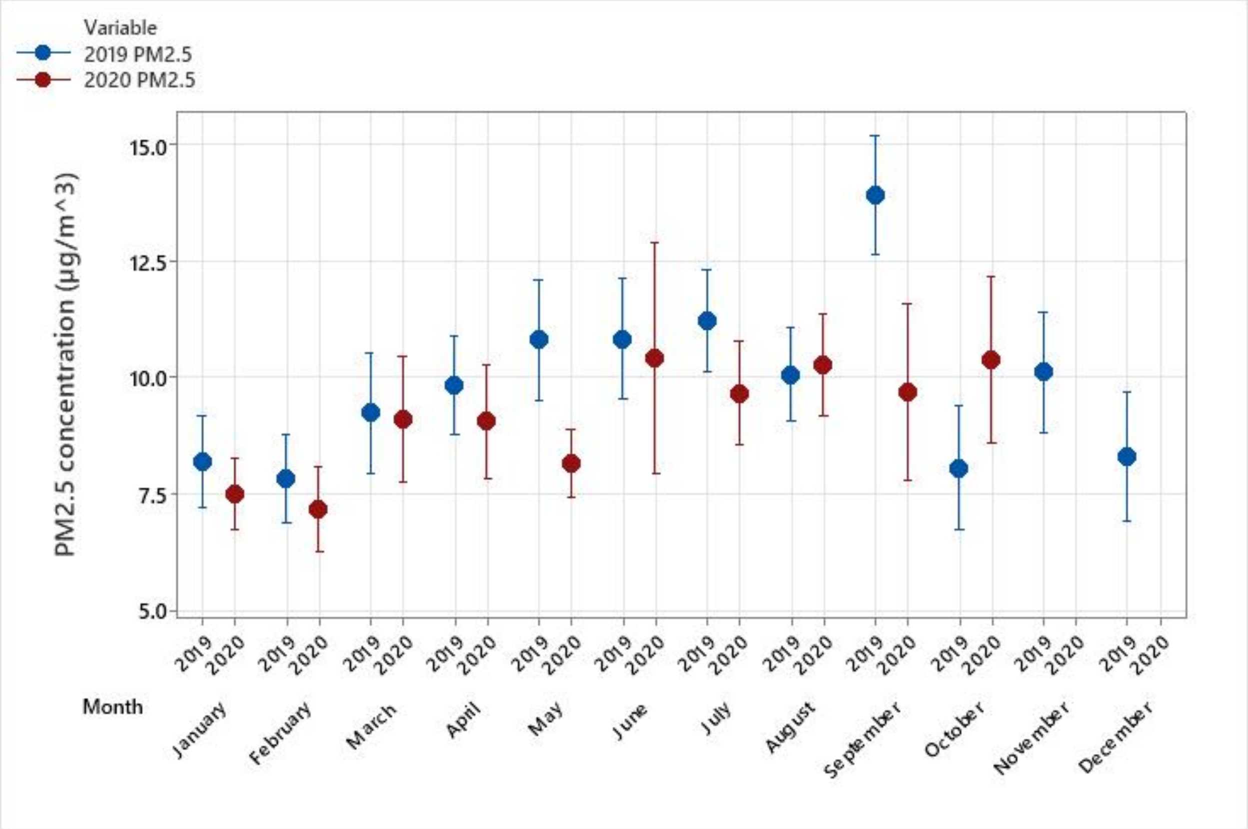

Figure S2: Monthly mean $PM_{2.5}$ concentrations (µg/m^3) in years 2019 and 2020. Error bars represent 95% confidence interval of the mean
